# Tuberculous severe acute colitis. A case report

**DOI:** 10.1016/j.amsu.2021.102756

**Published:** 2021-08-23

**Authors:** A. Haddad, A. Sebai, B. Chelly, H. Maghrebi, Y. Chaker, M. Jouini, M. Kacem

**Affiliations:** a Surgery Department A, La Rabta Hospital, Tunis, Tunisia; b Pathology Department, La Rabta Hospital, Tunis, Tunisia; c Faculty of Medicine of Tunis, Tunis El Manar University, Tunisia

**Keywords:** Tuberculosis, Severe acute colitis, Mycobacterium tuberculosis

## Abstract

**Introduction:**

and importance: Intestinal tuberculosis represents 2% of the ten million cases of tuberculosis reported in 2018. Herein, we report a case of tuberculous severe acute colitis. It is a rare and life-threatening condition. Our literature review found only five published cases. It occurs generally in immunocompromised patients. Extended colonic inflammation seems to be the main predictive factor of death. Moreover, an early diagnosis and rapid onset of antituberculous treatment are mandatory to save the patient's life.

**Case presentation:**

Herein, we present a case of tuberculous severe acute colitis with a review of the reported cases. The patient presented with a severe and idiopathic acute colitis. He was put on broad-spectrum antibiotics and intravenous corticosteroids. At day two, he developed septic shock and colic perforation. Colectomy was performed. Microbiology investigation and pathology examination confirmed tuberculous colitis.

**Clinical discussion:**

Tuberculous severe acute colitis occurs generally in immunocompromised patients. Extended colic inflammation seems to be the main predictive factor of death. Moreover, an early diagnosis and rapid onset of antituberculous treatment are mandatory to save the patient's life. However, diagnosis is difficult as symptoms aren't specific. Microbiology and pathology were compulsory to retain colic tuberculosis in all the reported cases.

**Conclusion:**

Tuberculous severe acute colitis is a challenging and life-threatening condition. It usually occurs in immunocompromised patients. Abdominal CT-scan may evoke the diagnosis. Microbiology and pathology are mandatory to retain the diagnosis. Early diagnosis and onset of antituberculous treatment are compulsory to save the patient's life.

## Highlights

• Diagnosis of tuberculous severe acute colitis is difficult.
• It usually occurs in immunocompromised patients.
• Surgery is mandatory if perforation occurs.
• Early diagnosis and onset of antituberculous treatment are compulsory to save the patient's life.

## 1 Introduction

In 2018, an estimated ten million tuberculosis cases were reported with only 2% of cases were related to intestinal tuberculosis [1]. It usually affects the final ileal loop, caecum, and ascending colon, and is a Crohn's disease-like [2]. Therefore, its diagnosis is challenging as symptoms aren't specific, making it a life-threatening condition. In our study we presented a case of tuberculous severe acute colitis as well as a review of the reported cases. This case report was reported according to SCARE criteria [3].

## 2 Case report

**Fig. 1:**
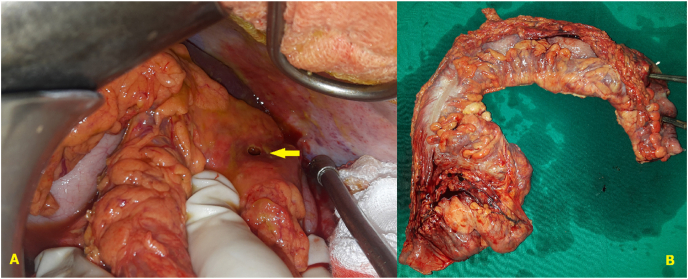
A) Peroperative view: splenic fixture's perforation. B) Specimen.

**Fig. 2:**
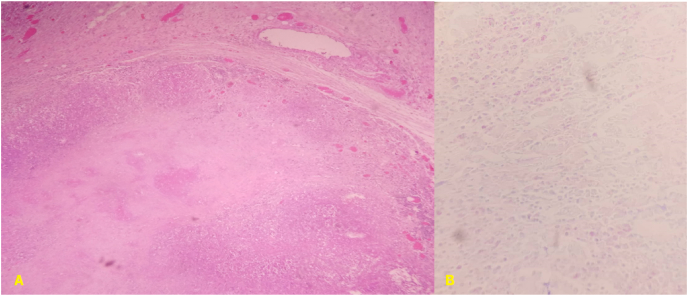
Pathology examination: A) Granulomatous inflammation with epithelioid macrophages, Langhans giant cells and characteristic caseous necrosis in the center (HES x 100). B) Ziehl-Neelsen stain showing acid fast bacilli in the necrotic tissue.

A 45-year-old man with no past medical history presented to our emergency department for acute abdominal pain, fever at 38,9 °C, chills and bloody diarrhea that lasted for two days. Before admission, he never experienced diarrhea nor bloody stools. He reported a recent weight loss of 7 kg in the last two weeks. Heart rate was at 100 beats/minute, blood pressure was at 110/70 mmHg, respiratory rate was at 20/minute. Abdominal examination found a diffuse tenderness without guarding. C reactive protein was at 40 mg/L. WBC was at 10200 cells/mm^3^. Haemoglobin was at 9 g/dL. Liver and renal functions were normal. Chest X-ray was normal. Colic diameter was normal on abdominal X-ray. On the emergency CT-scan, the colic and upper rectal walls were thickened and intensively enhanced after contrast fluid injection. There were also multiple necrotic lymph nodes and mesenteric hyperemia. There wasn't any intra-abdominal fluid or pneumoperitoneum nor sclerolipomatosis was found. Rectoscopy revealed large and deep ulcerations of the upper rectum with mucosal friability and bleeding and loss of the typical vascular pattern. Biopsies weren't possible to avoid iatrogenic colic perforation. The patient was put on broad-spectrum antibiotics and intravenous corticosteroids. At day two, he developed a septic shock and abdominal guarding. So, we decided to operate. We conducted a mid-line laparotomy and discovered a widespread purulent peritonitis with pseudomembranes. The colon and rectum were thickened, and the colon was perforated at the splenic fixture (Fig. 1). There also were multiple mesocolic lymph nodes measuring up to 1,5 cm with necrotic cheese-like material in the center. No additional digestive tract abnormalities or sclerolipomatosis were identified. We performed a subtotal colectomy with ileostomy and colostomy. The patient died at postoperative day two due to sepsis. The diagnosis of tuberculous colitis was confirmed by microbiological analysis of the free peritoneal fluid. Histologic examination of the specimen found a granulomatous inflammation with epithelioid macrophages and Langhans giant cells along with lymphocytes, plasma cells, fibroblasts with collagen, and characteristic caseous necrosis in the center. Ziehl-Neelsen stain shows the organisms as slender red rods (Fig. 2). Mycobacterium tuberculosis was identified using polymerase chain reaction.

## 3 Discussion

In this paper, we presented an uncommon presentation of tuberculosis: severe acute and isolated colitis. Tuberculosis affects the digestive tract in 2% of cases [1]. Usually, the last ileal loop, the caecum and the ascending colon are affected, imitating Crohn's disease. Reports of tuberculous severe acute colitis are uncommon. To the best of our knowledge, only five cases were published in the literature [[4], [5], [6], [7], [8]] (Table 1).

**Table 1:** The reported cases.

Case	Sex	Age	Immunocompromised	Involved segment of colon	Early onset of antituberculous treatment	Colic perforation	Death
*Charpentier & al, 1979* [4]	Male	49	Yes	Pancolitis	No	Yes	Yes
*Park & al, 2000* [5]	Male	49	Yes	Pancolitis	No	Yes	Yes
*Sikalias & al, 2009* [6]	Male	51	Yes	Right Colon	Yes	Yes	No
*Carkman & al, 2009* [7]	Male	39	Yes	Right colon	Yes	Yes	No
*Trad & al, 2018* [8]	Female	16	No	Caecum	Yes	No	No
*Our case*	Male	45	No	Pancolitis	No	Yes	Yes

Based on our review of published cases, we found that this condition is most common in immunocompromised patients. Extended colic inflammation seems to be the main predictive factor of death. Moreover, an early diagnosis and rapid onset of antituberculous treatment are mandatory to save the patient's life. However, diagnosis is difficult as symptoms aren't specific. Microbiology and pathology were compulsory to retain colic tuberculosis in all the reported cases. Presenting with an acute and idiopathic severe colitis, our patient was given intravenous corticosteroids. This contributed considerably to septic shock and colic perforation.

Retrospectively, we think that tuberculosis could have been evoked in our case. Indeed, abdominal CT-scan revealed multiple necrotic lymph nodes as usually seen in intestinal tuberculosis. Furthermore, the surgical specimen contained a necrotic cheese-like material in the center, which was suggestive of tuberculosis.

## 4 Conclusion

Tuberculous severe acute colitis is a challenging and life-threatening condition. It usually occurs in immunocompromised patients. Abdominal CT-scan might evoke the diagnosis. Microbiology and pathology are mandatory to retain the diagnosis. Early diagnosis and onset of antituberculous treatment are compulsory to save the patient's life.

## Funding source

No source has funded this manuscript.

## Ethical approval

All the authors have read and complied with the policy of the journal on ethical consent.

## Consent

Written informed consent was obtained from the patient for publication of this case report and accompanying images. A copy of the written consent is available for review by the Editor-in-Chief of this journal on request.

## Provenance and peer review

Not commissioned, externally peer-reviewed.

## Author contribution

Haddad Anis: Writing – original draft.

Sebai Amine: Writing – original draft.

Chelly Beya: Writing – original draft; Data interpretation of the pathological findings.

Maghrebi Houcine: Study concept and design.

Chaker Youssef: Methodology.

Montasser Kacem: Supervision.

## Registration of research studies

Not appliable.

## Guarantor

Sebai Amine.

## Declaration of competing interest

The authors declare that they have no conflict of interest.
